# Integrated Analysis to Study the Relationship between Tumor-Associated Selenoproteins: Focus on Prostate Cancer

**DOI:** 10.3390/ijms21186694

**Published:** 2020-09-13

**Authors:** Francesca Capone, Andrea Polo, Angela Sorice, Alfredo Budillon, Susan Costantini

**Affiliations:** Unità di Farmacologia Sperimentale-Laboratori di Mercogliano, Istituto Nazionale Tumori “Fondazione G. Pascale”—IRCCS, 80131 Napoli, Italy; f.capone@istitutotumori.na.it (F.C.); a.polo@istitutotumori.na.it (A.P.); a.sorice@istitutotumori.na.it (A.S.)

**Keywords:** selenoproteins, cancer, HUB nodes, prostate cancer

## Abstract

Selenoproteins are proteins that contain selenium within selenocysteine residues. To date, twenty-five mammalian selenoproteins have been identified; however, the functions of nearly half of these selenoproteins are unknown. Although alterations in selenoprotein expression and function have been suggested to play a role in cancer development and progression, few detailed studies have been carried out in this field. Network analyses and data mining of publicly available datasets on gene expression levels in different cancers, and the correlations with patient outcome, represent important tools to study the correlation between selenoproteins and other proteins present in the human interactome, and to determine whether altered selenoprotein expression is cancer type-specific, and/or correlated with cancer patient prognosis. Therefore, in the present study, we used bioinformatics approaches to (i) build up the network of interactions between twenty-five selenoproteins and identify the most inter-correlated proteins/genes, which are named HUB nodes; and (ii) analyze the correlation between selenoprotein gene expression and patient outcome in ten solid tumors. Then, considering the need to confirm by experimental approaches the correlations suggested by the bioinformatics analyses, we decided to evaluate the gene expression levels of the twenty-five selenoproteins and six HUB nodes in androgen receptor-positive (22RV1 and LNCaP) and androgen receptor–negative (DU145 and PC3) cell lines, compared to human nontransformed, and differentiated, prostate epithelial cells (EPN) by RT-qPCR analysis. This analysis confirmed that the combined evaluation of some selenoproteins and HUB nodes could have prognostic value and may improve patient outcome predictions.

## 1. Introduction

Selenoproteins are a class of proteins that contain selenium atoms inside selenocysteine (Sec) residues. Sec has been identified as the 21st amino acid, and is an analog of cysteine in which a selenol group replaces the sulfur-containing thiol group. It is encoded by the UGA codon that directs the translational decoding of UGA codons, rather than being used as a translational terminator. The corresponding mRNA includes a SEC Insertion Sequence (SECIS), which is present in eukaryotes in the 3′-untranslated region (UTR) of RNA [1].

Today, twenty-five selenoproteins have been identified in humans and twenty-four have been identified in mice. Mammalian selenoproteins can be classified mainly into two groups according to the Sec location. One group of selenoproteins possesses Sec at a site very close to the protein C terminus, and consists of three thioredoxin reductases (TXNRDs) and six additional proteins (methionine-R-sulfoxidereductase 1 (MSRB1), SELENOI, SELENOK, SELENOO, SELENOP, and SELENOS). The other group has Sec in the N-terminal part, and includes five glutathione peroxidases (GPX1, 2, 3, 4 and 6), three iodothyronine deiodinases (DIOs), and eight additional proteins (SELENOF, SELENOH, SELENOM, SELENON, SELENOT, SELENOV, SELENOW, and SEPHS2). These proteins are located in different sub-cellular compartments (nucleus, mitochondria, cytoskeleton, cytoplasm, endoplasmic reticulum (ER), Golgi apparatus and endosome). Some selenoproteins are secreted in the blood, such as SELENOP and GPX3 [2]. In addition, thioredoxin reductase 1 (TXRND1), a cytoplasmic and nuclear selenoprotein, can be secreted, and its serum levels are associated with poor prognosis in non-small cell lung cancer [3]. The majority of selenoproteins are involved in anti-oxidative activities associated with defending cells in different compartments against oxidative stress [4]. Some selenoproteins are located in the ER, and implicated in protein degradation, ER stress, and redox metabolism regulation [5]. These proteins are also involved in additional physiological functions, such as thyroid hormone metabolism, selenium transportation and storage, selenocysteine synthesis, protein folding, cell maintenance, calcium homeostasis, immune responses, and senescence [6]. Altered expression levels of selenoproteins have been associated with different disorders, such as type 2 diabetes, neuronal degenerative and cardiovascular diseases, and cancer [7,8].

Altered redox homeostasis can be involved in cancer initiation and progression because the oxidative insult can lead to genomic instability, DNA mutation, and carcinogenesis. Selenoprotein alteration has been reported to induce cancer initiation when associated with a low intake of selenium, thus increasing redox alterations. Indeed, several studies reported an inverse relationship between selenium levels and cancer risk. Recently, Lubiński et al. (2018) highlighted that in laryngeal cancer patients, the selenium level at the time of diagnosis was associated with the outcome [9]; similarly selenium supplementation in women undergoing treatment for breast cancer can favorably influence the patients’ outcomes [10]. Recently, two reviews highlighted the association between single selenoproteins and either colorectal or prostate cancer initiation and progression, as well as patient outcome, suggesting that these proteins could represent potential biomarkers or therapeutic targets [11,12]. Several additional reports also demonstrated the role of specific selenoproteins in other cancer types. High levels of SELENOM were associated with poor prognosis of renal cell carcinoma (RCC), and preclinical evidence demonstrated that SELENOM silencing was able to block cancer proliferation, invasion, migration, and tumorigenesis, via PI3K/AKt/mTOR pathway inhibition and reduced metalloproteinases (MMP) 2 and 9 expression [13]. SELENOK was found to have a crucial role in the proliferation and activation of immune cells and the promotion of calcium flux that induces melanoma progression [14]. SELENOS was found to be highly expressed in insulinoma cells, and its silencing is able to induce apoptosis by decreasing Bcl-xL in β-cells and to block the cell cycle by downregulating the transcription factor E2F1 and increasing cyclin dependent kinase inhibitor p27 expression [15].

Our group previously reported for the first time an increase of SELENOM and GPX4 expression in HCC liver tissues, and that their expression was associated with the malignancy grade [16,17]. Based on a transcriptomic and interactomic approach, a list of dysregulated selenoproteins was observed in two human HCC cell lines, HepG2 and Huh7, compared to normal human hepatocytes [18]. Moreover, we identified human miR-544a as able to modulate SELENOK expression in the two HepG2 and Huh7 cell lines [19], and also analyzed the selenoproteins’ transcriptomes in MCF-7 and MDA-MB231 human breast cancer cell lines, compared to MCF10A normal epithelial breast cells [20]. Recently, we reported that elevated tumor tissue expression of the selenoprotein SEPHS2 in triple-negative breast cancer (TNBC) patients was correlated with the malignancy grade [21].

Despite all the evidence reported above, the mechanism and the specific role of single or associated selenoproteins in cancer initiation and progression is still not clear. In silico approaches, such as bioinformatics, have been used to investigate signaling pathways as well as protein and gene interactions, in order to obtain a better understanding of the molecular mechanisms of diseases [22,23]. In detail, the construction of a gene/protein interaction network, and the application of a scoring algorithm based on the calculation of the quality, and the quantification, of interactions, followed by cluster analysis, might allow: (i) the selection of a list of genes/proteins putatively important in a given disease/cancer, according to the confidence and number of interactions derived from available databanks from experimental data; and (ii) the identification of “leader” genes/proteins that can be assumed to play important functional roles, because they present the highest number of interactions with the other genes/proteins within the network, thus being considered as HUB nodes in the interaction map [24,25,26,27]. It is noteworthy that the interaction networks comprise physical or functional correlations between all the genes/proteins involved in specific diseases/cancer types, indicated as direct or indirect interactions, respectively, and can be used to suggest the functional significance of the experimental results and clinical data that should be further confirmed in new targeted “wet” experiments. In this context, it can be useful to use bioinformatics approaches to identify associations between selenoprotein genes and proteins, and different cancer types.

Therefore, in the present study, publicly available datasets and different bioinformatics tools were used to, (i) analyze the protein–protein interactome of the twenty-five mammalian selenoproteins, and determine the most inter-correlated proteins, defined as HUB nodes; and (ii) correlate the gene expression, of the selenoproteins and the identified HUB nodes, in ten solid tumors, with patient outcomes.

Moreover, experimental approaches were used to confirm some of the correlations suggested by the bioinformatics analyses, based on a study on human prostate cancer cell lines. Indeed, within the last year, our group has focused on searching for new markers to predict prostate cancer initiation/progression [28,29] and improve the early definition of prostate cancer patient outcomes [30]. In detail, we evaluated the gene expression level of the twenty-five selenoproteins and the identified HUB nodes in prostate cancer cells, compared to normal epithelial prostate cells, by RT-qPCR analysis.

## 2. Results

### 2.1. Network Analysis

To better understand the relationships between selenoproteins and cancer, we conducted an interaction network analysis to define whether these proteins were inter-correlated, or correlated with other key proteins involved in cancer.

**Figure 1:**
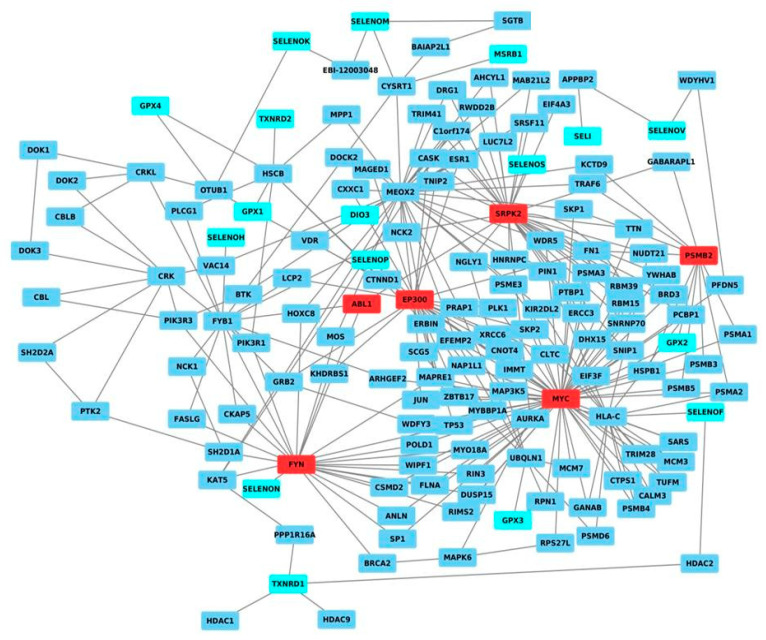
Network of the selenoproteins mapped on the human interactome. In detail, the selenoproteins are shown in cyan, HUB nodes are shown in red, whereas the other nodes are shown in blue.

Therefore, based on the human molecular interactome, we created an interaction network, in which the twenty-five known selenoproteins were the nodes, and the relationships between them were the edges. As shown in Figure 1, seventeen selenoproteins in the same network were correlated through different nodes, indicating functional redundancy and/or cooperation between different selenoproteins. To understand the positions and roles of the protein nodes, and identify the HUB nodes, which are the nodes with the strongest role in coordinating the network, the network was analyzed in terms of centrality and topological measures (node degree distribution, clustering coefficient, stress centrality, closeness centrality, betweenness centrality, clustering coefficient, network centralization, characteristic path length, average number of neighbors, network density, and network heterogeneity), as reported in the methods section. In detail, these statistical analyses showed that the obtained network had a network centralization value of 0.364, indicating that the network had good centralization, with the presence of nodes with a high degree. The portion of the potential connections into the network, expressed by the network density value, was equal to 0.023, whereas the higher network heterogeneity value was 1.825, suggesting a tendency of the network to contain HUB nodes. Moreover, Figure S1 shows the decreasing plots of the node degree distribution, clustering coefficient, stress centrality, closeness centrality, and betweenness centrality, which all together confirmed the scale-free property of the obtained network and its tendency to contain HUB nodes. These analyses identified the presence of the six following HUB nodes in the network: ABL1, EP300, FYN, MYC, PSMB2, and SRPK2.

The HUB nodes include the well-known oncogene MYC, which is amplified in various types of cancer, such as breast, colorectal, endometrial, ovarian, and prostate cancer, and controls the expression of genes and noncoding RNAs by regulating cell growth, cell cycle progression, apoptosis, and differentiation, as well as angiogenic switch, cellular metabolism, and drug resistance mechanisms [31,32]. Its expression has been correlated with prognostic/clinic-pathological outcomes in breast cancer [33], and occurs in the early stages of colorectal cancer [34].

EP300 has dual activities as a transcriptional factor and a histone acetyltransferase [35], and it is involved in different biological functions, such as proliferation, cell cycle regulation, apoptosis, differentiation, and DNA damage response [36]. EP300 gene has often been found to be mutated/truncated in lymphomas and different solid tumors, such as gastric, colorectal, breast, and pancreatic cancers, and over-expressed and correlated to poor prognosis in liver, nasopharyngeal, and small and non-small cell lung cancer [37].

FYN was reported to be correlated with cell motility and proliferation and over-expressed in chronic myeloid leukemia, breast cancer, squamous head and neck carcinoma, and melanoma [38]. In TNBC cells, FYN was shown to induce epithelial-mesenchymal transition (EMT), and its depletion reduced cell migration and invasion [39].

SRPK2 is part of a family of kinases that phosphorylate serine/arginine-rich (SR) proteins and regulate the conformation, subcellular localization, and/or interaction of SR enzymes, to regulate posttranscriptional mRNA processing [40]. In a colon cancer model, this protein was demonstrated to promote cell growth and migration, and control the expression of lipogenic enzymes [41].

ABL1 is a tyrosine kinase involved in different cellular signaling processes, controlling proliferation, survival, migration, and invasion of cells [42]. It was initially identified as a tumor suppressor but, since then, its oncogenic functions have been well-established [42]. However, data in solid tumors suggest that ABL1 can have tumor suppressive or oncogenic roles, depending on the cellular context.

PSMB2 is a component of the proteasome complex involved in the degradation of intracellular proteins, playing a role in maintaining protein homeostasis [43]. It was significantly associated with chronic leukemia [44], and its suppression was able to inhibit liver cancer proliferation [45]. Moreover, in our previous paper, PSMB2 was a HUB node in the bladder cancer network [46].

Overall, our network analysis can represent a starting point for planning targeted experiments, for verifying the correlations among selenoproteins (in terms of gene expression levels and/or functions), and with these HUB nodes, in different types of cancer via experimental approaches.

### 2.2. Correlation between Selenoproteins/HUB Nodes and Cancer Patient Overall Survival

To highlight eventual specific relationships between selenoproteins and cancers, we evaluated the correlation between the gene expression of the twenty-five selenoproteins and the overall survival (OS) of patients, for the ten most common solid tumor types, using different public datasets and the PROGgeneV2 online tool (http://genomics.jefferson.edu/proggene/index.php) [47].

**Figure 2:**
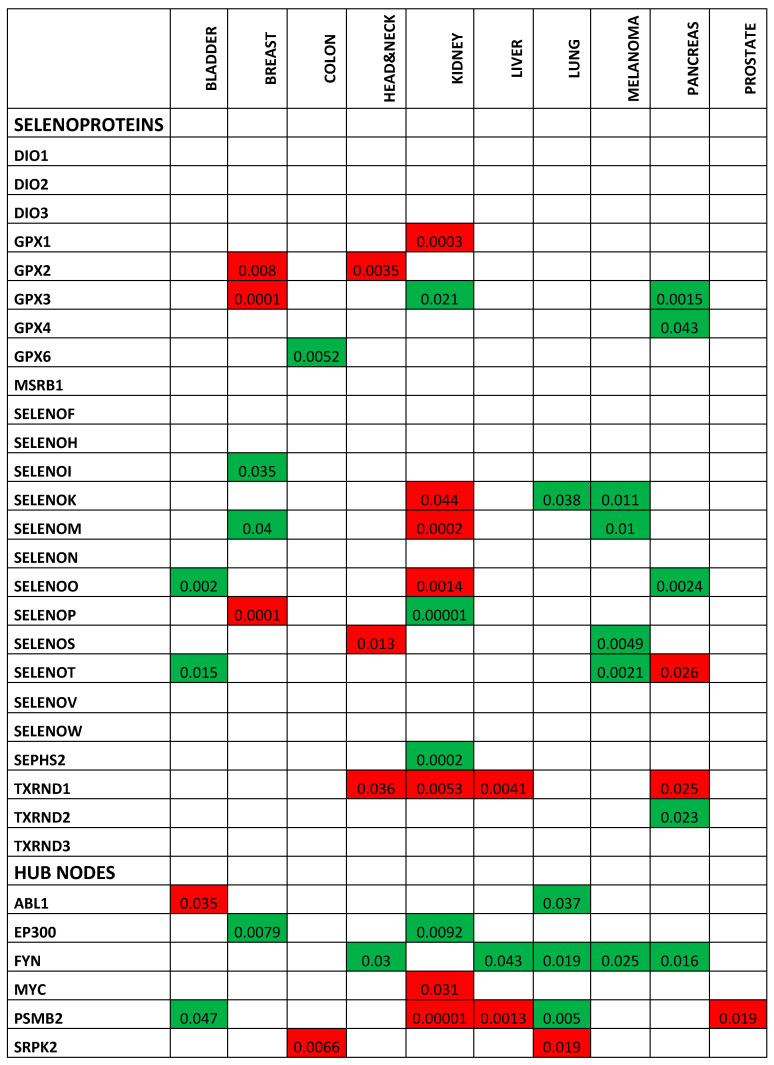
Heat map related to the statistically significant correlations (with *p*-values < 0.05) between overall survival (OS) in solid tumor patients and selenoprotein/HUB node expression. In detail, we report significant *p*-values in red or in green, if the high or low expression of selenoproteins, was correlated with poor overall survival, respectively. White boxes indicate no significant correlations.

**Figure 3:**
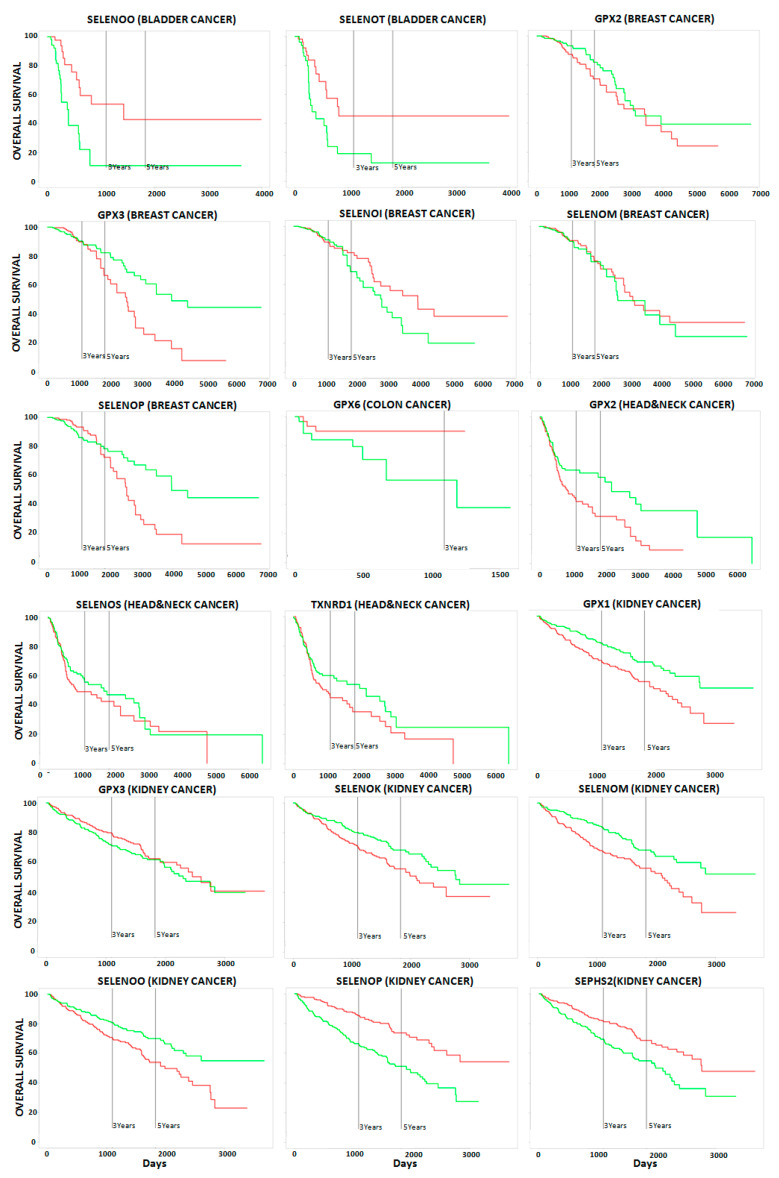

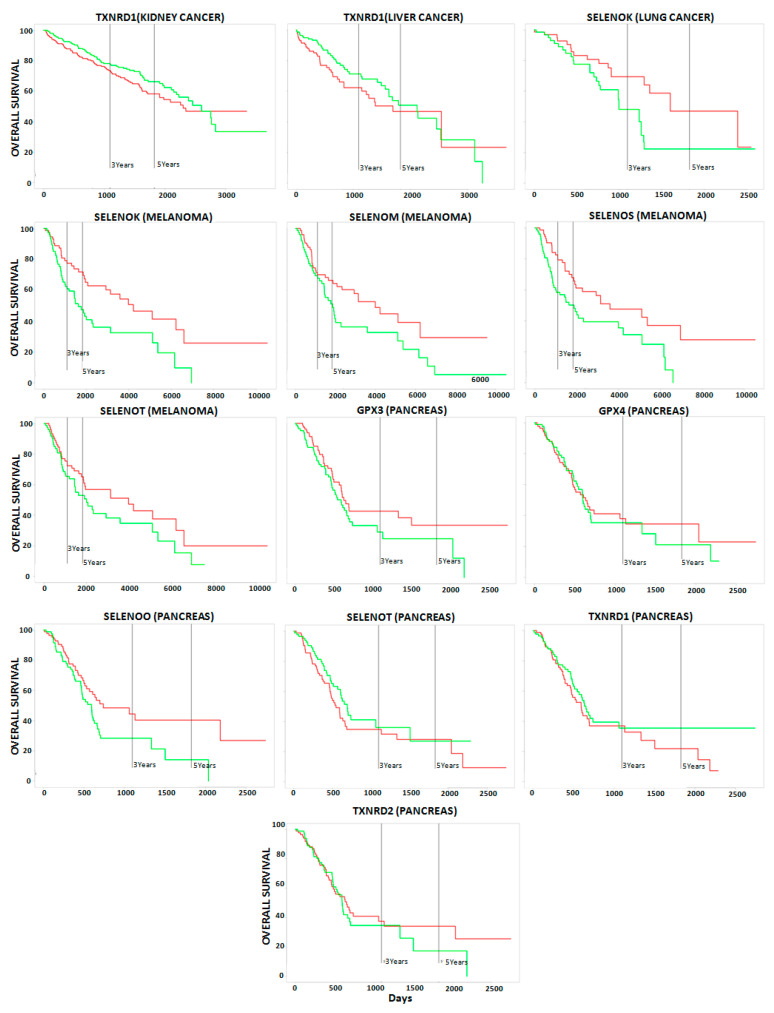
Kaplan-Mayer curves showing the overall survival (expressed in percentage) in solid cancer patients using the PROGgeneV2 online tool, in the case of high and low expression of selenoproteins, which are indicated by red and green curves, respectively.

This analysis demonstrated that (i) no correlation occurred between single selenoprotein expression and poor OS in the case of prostate cancer patients; (ii) no correlation occurred between ten selenoproteins (DIO1, DIO2, DIO3, MSRB1, SELENOF, SELENOH, SELENON, SELENOV, SELENOW, and TXNRD3) and poor OS in any of the ten analyzed cancer types; and iii) a statistically significant correlation occurred between the expression levels of the fifteen selenoproteins with poor OS in at least one cancer type (Figure 2 and Figure 3).The largest number of selenoproteins with altered gene expression, either upregulation or downregulation, in tumor tissues compared with normal tissues, and correlations with poor OS, were found in kidney (eight), pancreas (six), and breast (five), cancers. The expression of some selenoproteins was altered in more than one cancer type; however, in the majority of cases, different patterns of selenoprotein gene expression were observed among the cancers examined. The only exceptions were (i) GPX2, whose high expression levels correlated with OS in breast and head and neck cancers; and (ii) TXNRD1, whose high expression levels correlated with poor OS in head and neck, kidney, liver, and pancreas cancers.

To analyze in more detail the correlation between the identified HUB nodes and the selenoproteins in ten examined cancers, we evaluated the correlation between HUB node expression and OS, in patients with these ten cancers. As shown in Figure 2 and Figure S2, all six HUB nodes correlated with at least one cancer type, and FYN and PSMB2 correlated with poor OS in patients with five different cancers. In particular, lower expression levels of FYN correlated with poor OS in patients with melanoma, head and neck, liver, lung, and pancreas cancers. On the other hand, higher expression levels of PSMB2 were correlated with poor OS in patients with kidney, liver, and prostate cancers, whereas lower levels of PSMB2 were correlated with poor OS in patients with bladder and lung cancers. Hence, in the case of prostate cancer, we did not observe a correlation between single selenoprotein expression and patient OS, and a significant correlation was only observed between the HUB node PSMB2 and poor OS.

### 2.3. Gene Expression Levels of Twenty-Five Selenoproteins in Prostate Cancer Cells Compared to Normal Prostate Cells

Next, to experimentally evaluate the data suggested by the bioinformatics analyses, and better define the selenoprotein alterations in prostate cancer as well as the correlation between them and the HUB nodes, we conducted a preliminary study on gene expression in five cell lines. In detail, the gene expression profiles for all twenty-five selenoproteins, in two androgen receptor-positive cell lines (22RV1 and LNCaP), and two androgen receptor-negative cell lines (DU145 and PC3), were compared to human nontransformed, and differentiated, prostate epithelial cells (EPN), based on a RT-qPCR assay.

**Figure 4:**
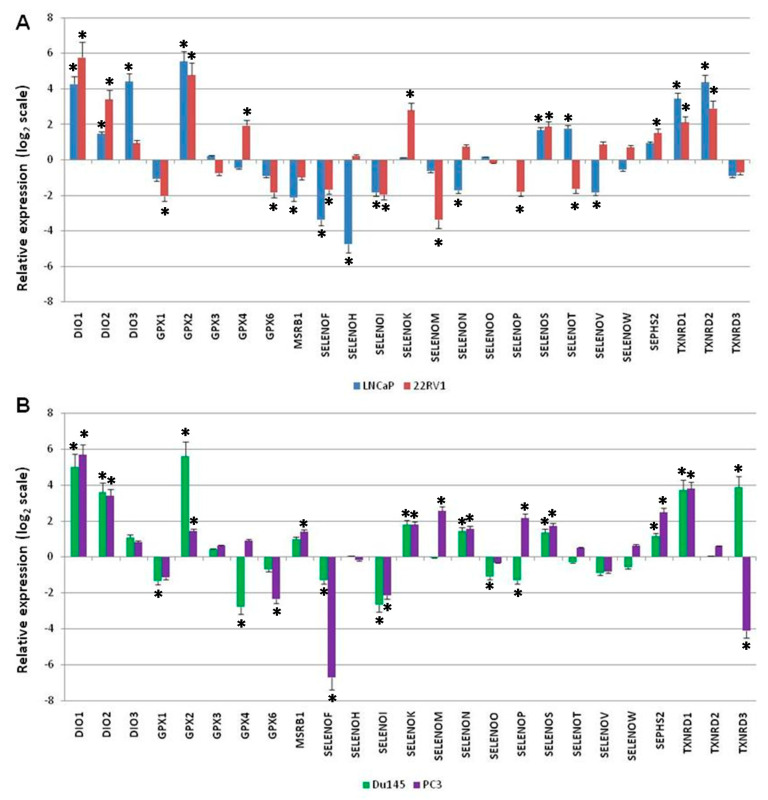
Fold change of gene expression level for each selenoprotein (indicated as the relative expression) in (**A**) two androgen receptor-positive (LNCaP and 22RV1) and (**B**) two androgen receptor-negative (DU145 and PC3) prostate cancer line cells, compared to the non-cancerous epithelial cells (EPN) cells, as evaluated by the 2^−ΔΔCq^ method, and reported on the log_2_ scale. We considered values higher and lower than +1 and −1 to be statistically significant, respectively. The statistically significant *p*-values at <0.05 are indicated by *.

The results revealed, for the two androgen receptor-positive cell lines (Figure 4A), (i) a statistically significant increase (with log_2_ 2^−ΔΔCt^ > 1) of the expression levels of DIO1, DIO2, GPX2, SELENOS, TXNRD1, and TXNRD2; and ii) a statistically significant decrease (with log_2_ 2^−ΔΔCt^ < 1) of the expression levels of SELENOF and SELENOI. Compared to EPN cells, LNCaP cells showed higher DIO3 and SELENOT levels, but lower MSRB1, SELENOH, SELENON, and SELENOV levels. Compared to EPN cells, 22RV1 cells showed higher expression levels of GPX4, SELENOK, and SEPHS2, and lower levels of GPX1, GPX6, SELENOM, SELENOP, and SELENOT.

As shown in Figure 4B, the two androgen receptor-negative cell lines showed: (i) a statistically significant increase (with log_2_ 2^-ΔΔCt^ > 1) of the expression levels of DIO1, DIO2, GPX2, SELENOK, SELENON, SELENOS, SEPHS2, and TXNRD1; and (ii) a statistically significant decrease (with log_2_ 2^-ΔΔCt^ < 1) of the expression levels of SELENOF and SELENOI. Moreover, compared to EPN cells, DU145 cells showed higher levels of TXNRD3 and lower levels of GPX1, GPX4, SELENOO, and SELENOP, whereas PC3 cells showed higher levels of MSRB1, SELENOM, and SELENOP, and lower levels of GPX6 and TXNRD3.

### 2.4. Gene Expression Levels of HUB Nodes in Prostate Cancer Cells, and Their Correlation with Selenoprotein Expression

**Figure 5:**
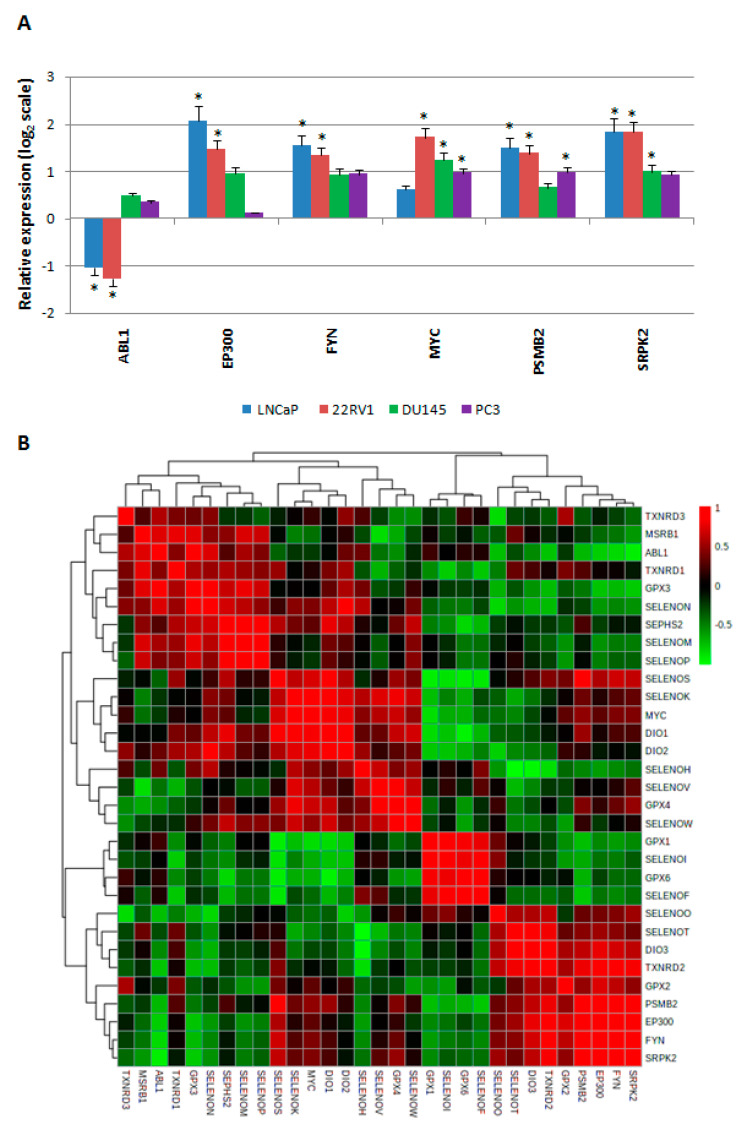
Evaluation of the gene expression levels of HUB nodes in five prostate cell lines and their correlation with the gene expression profiles of the selenoproteins. (**A**) Fold change of gene expression levels for each HUB node, in two androgen receptor-positive (LNCaP and 22RV1), and in two androgen receptor-negative (DU145 and PC3) prostate cancer line cells, compared to the non-cancerous EPN cells, as evaluated by the 2^−ΔΔCq^ method and reported as the log_2_ scale. We considered values higher and lower than +1 and −1 to be statistically significant, respectively. The statistically significant *p*-values at <0.05 are indicated by *. (**B**) Pearson correlation matrix evaluated on the gene expression profiles of the selenoproteins and HUB nodes, evaluated for EPN and prostate cancer cells. Color scale, from red to green color, indicates from good to poor correlations between the gene expression levels of the analyzed proteins.

Then, we evaluated the gene expression profiles for the six HUB nodes (ABL1, EP300, FYN, MYC, PSMB2, and SRPK2) in all the prostate cancer cells, compared to normal prostate EPN cells (Figure 5A).

These analyses showed that (i) ABL1 had lower statistically significant levels (with log_2_ 2^−ΔΔCt^ < −1) in both androgen receptor-positive cell lines (22RV1 and LNCaP), but higher levels in the two androgen receptor-negative cell lines, although these values were not statistically significant; (ii) the EP300, FYN, MYC, PSMB2, and SRPK2 levels increased in all four prostate cancer cell lines, although the EP300 and FYN levels were not statistically significant. In both androgen receptor-negative cells, the MYC levels were not significant in the LNCaP cells, the PSMB2 levels were not significant in the DU145 cells, and the SRPK2 levels were not significant in the PC3 cells.

To determine whether a correlation occurred between the gene expression levels of all HUB nodes and the selenoproteins evaluated, we performed a Pearson correlation matrix analysis. Figure 5B shows that (i) MYC and SELENOK were very correlated with each other and with DIO1, DIO2, and SELENOS; (ii) ABL1 showed a slight correlation with MSRB1; and (iii) EP300, FYN, SRPK2, and PSMB2 were correlated with GPX2.

**Figure 6:**
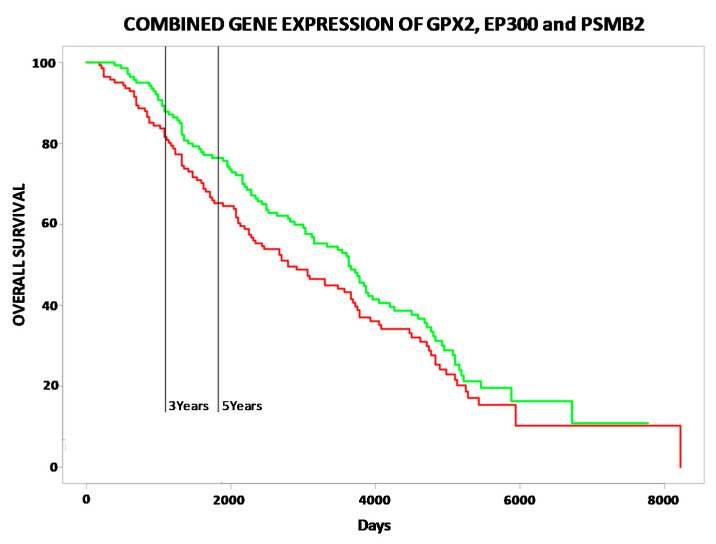
Kaplan-Mayer curves showing the correlations between overall survival (expressed in percentage) and the combined gene expression of GPX2, EP300, and PSMB2 in prostate cancer patients, which was based on the PROGgeneV2 online tool. High and low expression of selenoproteins are reported by the red and green curves, respectively.

Therefore, these data confirmed the correlation between selenoproteins and HUB nodes in prostate cancer cells, and suggested the need to conduct more detailed studies to analyze the putative role of these proteins as combined markers of progression or response to therapeutic approaches in prostate cancer. In this regard, we preliminarily evaluated whether significant correlations occurred between the combined gene expression of selenoproteins and HUB nodes (based on clustering in the matrix analysis shown in Figure 5B), and the OS of prostate cancer patients; this analysis was performed using the PROGgeneV2 online tool (http://genomics.jefferson.edu/proggene/index.php) [47]. Furthermore, this analysis highlighted a statistically significant correlation (with *p*-value = 0.0091) between the combined gene expression of GPX2, EP300, and PSMB2 (Figure 6), and the OS of prostate cancer patients, thus confirming that the combined evaluation of some selenoproteins and HUB nodes, could represent a new strategy to predict patient outcomes.

## 3. Discussion

The selenoprotein family has a well-established function in regulating oxidative cell balance [48], and is involved in tumorigenesis and cancer progression [6]. Still, identifying tumor type-specific selenoprotein profiles, and determining whether these proteins can predict prognosis or could serve as therapeutic anticancer targets in cancer patients, represent critical challenges. In this study, we created an interaction network of the twenty-five known selenoproteins and highlighted the presence of six HUB nodes (ABL1, EP300, FYN, MYC, PSMB2, and SRPK2) that play the strongest role in coordinating the obtained network. Then, we evaluated the correlation between the gene expression of the twenty-five selenoproteins and HUB nodes, and the OS of patients with the ten most common solid tumor types, with the results demonstrating that (i) ten selenoproteins were not correlated with poor OS of cancer patients; (ii) more correlations between selenoprotein gene expression and patient OS were only observed for kidney, pancreas, and breast cancers; (iii) single selenoprotein expression and OS of prostate cancer patients was not observed; and (iv) all HUB nodes were correlated with poor OS for at least one cancer type.

To our knowledge, few selenoproteins have been studied in prostate cancer, and never altogether. For example, GPX1, SELENOF, and SELENOP levels were significantly reduced in prostate cancer models [49,50,51], whereas GPX2 was significantly increased [52]. Increased nuclear TXNRD1 levels were found in high-grade, versus low-grade, human prostate cancers [53], and correlated with prostate cancer progression and androgen-deprived castration-resistant prostate cancer (CRPC) cells, suggesting that CRPC possesses an enhanced dependency on TXNRD1 [54]. Hence, our study aimed to confirm, by experimental approaches, some of the correlations suggested by bioinformatics analyses, and, thus, represented the first systematic evaluation of the expression levels of all twenty-five selenoproteins in androgen receptor-positive and -negative prostate cancer cells. Through our analysis, we confirmed higher levels of GPX2 and TXNRD1, and lower levels of GPX1, SELENOF, and SELENOP in the cancer cell lines compared to normal epithelial cells, which has already been observed and reported in the literature. We also observed higher levels of DIO1, DIO2, and SELENOS, and lower levels of SELENOI in all the prostate cancer cell lines. The differences observed between androgen receptor-negative or androgen receptor-positive cell lines can also be correlated with their different molecular features [55,56,57]. Although the number of tested cell lines was small, the differences between the androgen receptor-positive and androgen receptor-negative cell lines were not highlighted in detail; rather, we only suggested that our analyses showed a different expression pattern for some selenoproteins in prostate cancer compared to normal prostate epithelium. This observation should be functionally evaluated and eventually expanded to a greater number of prostate cancer lines and prostate tumor tissues; however, this task is beyond the scope of the current study.

Still, an interesting point of discussion is represented by the finding of lower levels of SELENOI in prostate cancer cells vs. EPN. SELENOI, also known as ethanolphosphotransferase 1 (EPT1), is an enzyme responsible for the final step in the Kennedy pathway that transfers phosphoethanolamine from cytidine diphosphate ethanolamine to lipid acceptors, to produce ethanolamine glycerophospholipids, such as phosphatidylethanolamine (PE). Hence, it plays an important role in maintaining the normal homeostasis of ether-linked phospholipids in humans [58]. Interestingly, an analysis of the concentrations of plasma phospholipids in prostate cancer patients showed that the levels of phosphatidylethanolamine, and total phospholipids in these patients, were decreased, and correlated with an increased pathologic grade and Gleason score [59]. We can then speculate that the lower levels of SELENOI found in prostate cancer cells could be correlated to decreased levels of phospholipids, and could represent a new putative marker of prostate cancer progression that warrants further study.

Moreover, as reported above, in our study we evaluated the gene expression levels of HUB nodes and performed a correlation with the expression of the selenoproteins by correlation matrix analysis (Figure 5B). We observed that: (i) MYC and SELENOK expression was strongly correlated with each other, and with DIO1, DIO2, and SELENOS; (ii) ABL1 showed a slight correlation with MSRB1; and (iii) EP300, FYN, SRPK2, and PSMB2 were correlated with GPX2.

Considering the first correlation cluster, SELENOK and SELENOS are two ER selenoproteins that regulate ER stress and degradation [60]. Both of these proteins have a role in the ER-associated protein degradation (ERAD) pathway, and interact with the valosin containing protein (VCP/p97) for the retrotranslocation of misfolded proteins, from ER to the cytosol, via their polyubiquitination, and they also play a role in cell survival [61,62,63]. Moreover, SELENOK showed peroxidase activity capable of reducing harmful hydrophobic substrates, such as phospholipid hydroperoxides, and is involved in membrane repair [64]. SELENOK was also found to be over-expressed in gastric, glial, thyroid, testis, and cervix cancers, and its polymorphisms, in combination with selenium status, were related to prostate cancer progression [65,66]. Recently, we showed that SELENOK was over-expressed in two liver cancer cell lines, HepG2 and Huh7 [18]. Furthermore, SELENOK inhibition by miR-181 and miR-544a was able to suppress the proliferation of glioma and hepatocellular carcinoma cells, respectively [19,67]. Regarding SELENOS, its polymorphisms were associated with many tumors, such as colorectal and gastric cancer [68], and were able to affect the expression levels of inflammatory cytokines in plasma [69]. On the other hand, DIO1 and DIO2 are located on the plasma membrane and ER, respectively, and are part of the iodothyronine deiodinase family and involved in regulating the activity of thyroid hormones by deiodination reactions [61]. DIO2 was found to be highly expressed in mesothelioma cell lines [70], and its inhibition resulted in the suppressed expression of prostate specific antigen (PSA) in prostate cancer, thus representing a potential approach to overcoming castration resistance [71]. Moreover, in prostate cancer, MYC activity was correlated with dysregulation of the PI3K/AKT/mTOR pathway, which induced cellular survival. The therapeutic efficacy of targeting MYC activity by interfering with its transcriptional program was also evaluated [72]. Detailed data about the involvement of DIO1, SELENOK, and SELENOS in prostate cancer have not been available until now; thus, additional data on these selenoproteins must be obtained to confirm their putative role in prostate cancer and their possible functional correlation with MYC.

In the second correlation cluster, MSRB1 is a selenoprotein located in the cytosol and the nucleus. It is mainly known for its antioxidant and protein repair functions and its role as a switch for protein function, via reversible oxidation/reduction of specific methionine residues [73,74]. MSRB1 contributes to shaping cellular immune responses, and its silencing resulted in the induction of anti-inflammatory cytokines (IL-10 and IL-1ra) [75]. High levels of MSRB1 were previously found in hepatocellular carcinoma, and correlated with the MAPK pathway and epithelial-mesenchymal transition (EMT) [76], and in low metastatic MCF7 human breast cancer cells [77]. ABL1 has been previously reported to act as a switch between cellular, invasive and proliferative, states and can either promote invasion and prostate cancer aggressiveness, or inhibit its progression, depending on the signal [78]. Hence, as in the case of the first cluster, it will be interesting to plan further studies to analyze the correlation between MSRB1 and ABL1.

Finally, in the third correlation cluster, we found that GPX2 was correlated with four HUB nodes (EP300, FYN, SRPK2, and PSMB2). GPX2, a key molecule of the glutathione redox system that acts in concert to provide a coordinated network of protection against ROS accumulation and oxidative damage, has been suggested as a prognostic marker in CRPC, and its silencing is able to inhibit prostate cancer growth [52]. Recently, high levels of EP300 were correlated to both prostate cancer progression and chemotherapy resistance in metastatic CRPC patients, and it has emerged as a possible co-target in chemo-resistant prostate cancer treatment [79]. FYN over-expression was demonstrated in prostate cancer and suggested as an interesting therapeutic target [80]. Moreover, SRPK2 and PSMB2 over-expression was observed in prostate cancer, and correlated with a high Gleason score, advanced pathological stage, tumor metastasis [81], and significantly poor 10-year metastatic rate in prostate cancer, in younger men [82]. Overall, these data are consistent with the significant correlation found between the combined gene expression of GPX2, EP300, and PSMB2, and OS in prostate cancer patients (Figure 6). These observations confirm that the combined evaluation of some selenoproteins and HUB nodes could have a better prognostic value, and could improve the prediction of patient outcomes.

## 4. Materials and Methods

### 4.1. Network Analysis

Through the Cytoscape software platform, for the visualization of complex networks and their integration (http://www.cytoscape.org/), a network related to the interactions between the selenoproteins was constructed, and used as reference for the human molecular interactome (INTACT), where all interactions are derived from literature curation or direct user submissions. As already reported in our recent paper, some statistical analyses on the following centrality and topological measure parameters of networks were performed: node degree distribution, clustering coefficient, stress centrality, closeness centrality, betweenness centrality, clustering coefficient, network centralization, characteristic path length, avg. number of neighbors, network density, and network heterogeneity [46]. The obtained network has been deposited in the NDEX database (http://www.ndexbio.org/#/network/194d2011-7c7a-11e9-848d-0ac135e8bacf?accesskey=d37870a03d06010b5b49c17e5411056441c9e7bac99ddde7d7354c8f4c68fd11).

### 4.2. Survival Gene Analysis

Using the PROGgeneV2 online tool (http://genomics.jefferson.edu/proggene/index.php) [47], we evaluated the correlation between selenoproteins/HUB nodes gene expression and OS in the ten most common solid tumor types, using different public datasets. The following ten datasets were used to evaluate the correlation between selenoprotein gene expression and OS: TCGA_COAD (Colon Adenocarcinoma), TCGA_PAAD (Pancreatic Adenocarcinoma), TCGA_SKCM (Skin cutaneous melanoma), TCGA_LIHC (Liver hepatocellular carcinoma), TCGA_PRAD/GSE16560 (Prostate Cancer), TCGA_BRCA (Breast Cancer), TCGA_HNSC (Head and Neck squamous cell carcinoma), TCGA-LUAD (Lung adenocarcinoma), TCGA_KIRC (Kidney renal clear cell carcinoma), and TCGA_BLCA (Bladder Urothelial Carcinoma).

### 4.3. Prostate Cancer Cell Lines

The expression of all twenty-five selenoproteins and HUB nodes was investigated by RT-qPCR in an EPN line, two androgen receptor-positive cell lines (22RV1 and LNCaP), and two androgen receptor-negative cell lines (DU145 and PC3).

EPN cells are a novel epithelial cell line derived from human prostate tissue that does not form colonies in semisolid medium and does not form tumors once injected into nude mice. They express the functional androgen receptor cytokeratin (numbered 1, 5, 10, and 14) and have wild-type p53 [83]. EPN cells were grown in DMEM/Ham’s F-12 50/50 supplemented with fetal bovine serum (5%) (Invitrogen, CA, USA), penicillin/streptomycin (100×) (Euroclone, Devon, UK), and Glutamax (100×) (Invitrogen, CA, USA).

22RV1, DU145, LNCaP, and PC3cells were grown in RPMI (Roswell Park Memorial Institute) supplemented with fetal bovine serum (Invitrogen, CA, USA) (10%), penicillin/streptomycin (100×) (Euroclone, Devon, UK), and Glutamax (100×) (Invitrogen, CA, USA).

### 4.4. RNA Preparation and Reverse Transcription-qPCR (RT-qPCR)

Total RNA was extracted from five prostate cell cultures (EPN, 22RV1, DU145, LNCaP, and PC3), using a RNAeasy Mini Kit (Qiagen Inc.) according to the manufacturer’s instructions. The RNA concentration and purity were determined using a NanoDrop 2000 spectrophotometer (Thermo Scientific, Wilmington, DE, USA) at an optical density of 260/280 nm. Reverse transcription of RNA was performed with 2 μg of RNA using a SuperScript VILO cDNA Synthesis kit (Life Technologies) in a 20 μL reaction volume.

The mRNA sequence from the nucleotide data bank (NCBI, Bethesda, Maryland, USA) was used to design primer pairs for RT-qPCR with an amplicon <100 bp, according to the manufacturer’s instructions. Oligonucleotides were obtained from Eurofins. The list of primers is reported in Table S1. RT-qPCR experiments were performed using a Step-One Real Time PCR System (Applied Biosystems). Each aliquot of cDNA (2 µL) was amplified in a mixture (25 µL) consisting of the reverse and forward primers (300 nM) and 2X SYBR Green PCR Master Mix (Applied Biosystems). The PCR conditions consisted of an initial denaturation step of 95 °C for 5 min followed by 44 cycles of a two-step program: (i) denaturation at 95 °C for 30 s; and (ii) annealing/extension at 60 °C for 1 min. Each assay included a no-template control for each primer pair, and to ensure that RNA samples were not contaminated with DNA, negative controls were obtained by performing the PCR assay on samples that were not reverse transcribed. Moreover, experiments were performed in triplicate to ensure reproducibility of the technique. β-Actin mRNA was used to normalize the data. All of the obtained data were analyzed statistically.

Sample ΔCq values were calculated as the difference between the mean Cq obtained for each selenoprotein transcript (seleno-mRNA) and the housekeeping gene. 2^−ΔΔCq^ values were determined to define the fold change of expression level for each seleno-mRNA in different prostate cancer cells compared to the non-cancerous EPN cells. We reported values on the log_2_ scale, and considered values higher and lower than +1 and −1 to be statistically significant, respectively. The statistical comparison between gene expression levels evaluated in the prostate cancer cell line vs EPN cells was calculated by the T-test. *P*-values lower than 0.05 were considered statistically significant.

Moreover, a Pearson correlation matrix analysis, comparing the gene expression profiles of the selenoproteins and HUB nodes evaluated for each prostate cancer cell line and the EPN cell line, was performed using the MetaboAnalyst tool (https://www.metaboanalyst.ca/).

## 5. Conclusions and Future Perspectives

In conclusion, our data add new insights into the cancer biology of selenoproteins, suggesting that further experimental studies are necessary for a deeper understanding of the role of these proteins, and the correlated hub nodes, as potential cancer prognostic markers and/or therapeutic targets. Certainly, because the members of the selenoprotein family are strongly interconnected, only a systematic approach should be used to obtain a global vision of the relationships between these proteins and cancer. We are aware that our approach has limitations, such as the potential risk of circular reasoning due to the lack of localization, and function annotations that are often incomplete or unavailable, since the interactomes of many species are unmapped. Hence, to improve the data quality in the interaction networks, it would be useful to analyze the properties of interacting gene/protein pairs, and to verify that they have similar process and functional annotations, common sub-cellular localizations, and shared interaction partners [84]. Moreover, experimental validation of the proteins/genes evidenced by in silico studies is always necessary, because these data must be considered as exploratory and used as a source of experimental and clinical hypotheses [23].

Indeed, using prostate cancer as a study model, our results suggest that some selenoproteins, such as MSRB1, SELENOI, SELENOK, SELENOS, and GPX2 (in particular) can represent interesting topics for further studies, being those proteins that are involved in inflammatory and metabolic/lipidic pathways, correlated with HUB nodes, and involved in prostate cancer. Moreover, whether these proteins are measurable in biological fluids, such blood, urine and saliva, should be verified, because they could represent ideal new biomarkers for the dynamic monitoring of prostate cancer progression and therapeutic approach responses.

Collectively, we confirmed that the role of all selenoproteins, rather than single members of the family, should be further evaluated in specific cancer types. To that end, our group is currently evaluating the expression of all twenty-five selenoproteins, at the mRNA and protein levels, in a group of selected breast cancer cell lines and tissues (manuscripts in preparation).

## Supplementary Materials

Supplementary materials can be found at https://www.mdpi.com/1422-0067/21/18/6694/s1. Figure S1. Evaluation of topological properties of network. (A) node degree distribution, (B) average clustering coefficient, (C) stress centrality, (D) closeness cenytrality and (E) betweenness centrality measure. Figure S2. Kaplan-Mayer curves showing the overall survival (expressed in percentage) in solid cancer patients, using PROGgeneV2 online tool, in the case of high and low expression of HUB nodes reported by red and green curves, respectively. Table S1. List of primer sequences
