# Supplementary material for: Integrated Analysis to Study the Relationship between Tumor-Associated Selenoproteins: Focus on Prostate Cancer
Source: Int J Mol Sci. 2020 Sep 13;21(18):6694. doi: 10.3390/ijms21186694 (PMC7555134; doi:10.3390/ijms21186694)
Supplement: Supplementary file 1 [file ijms-21-06694-s001.pdf]

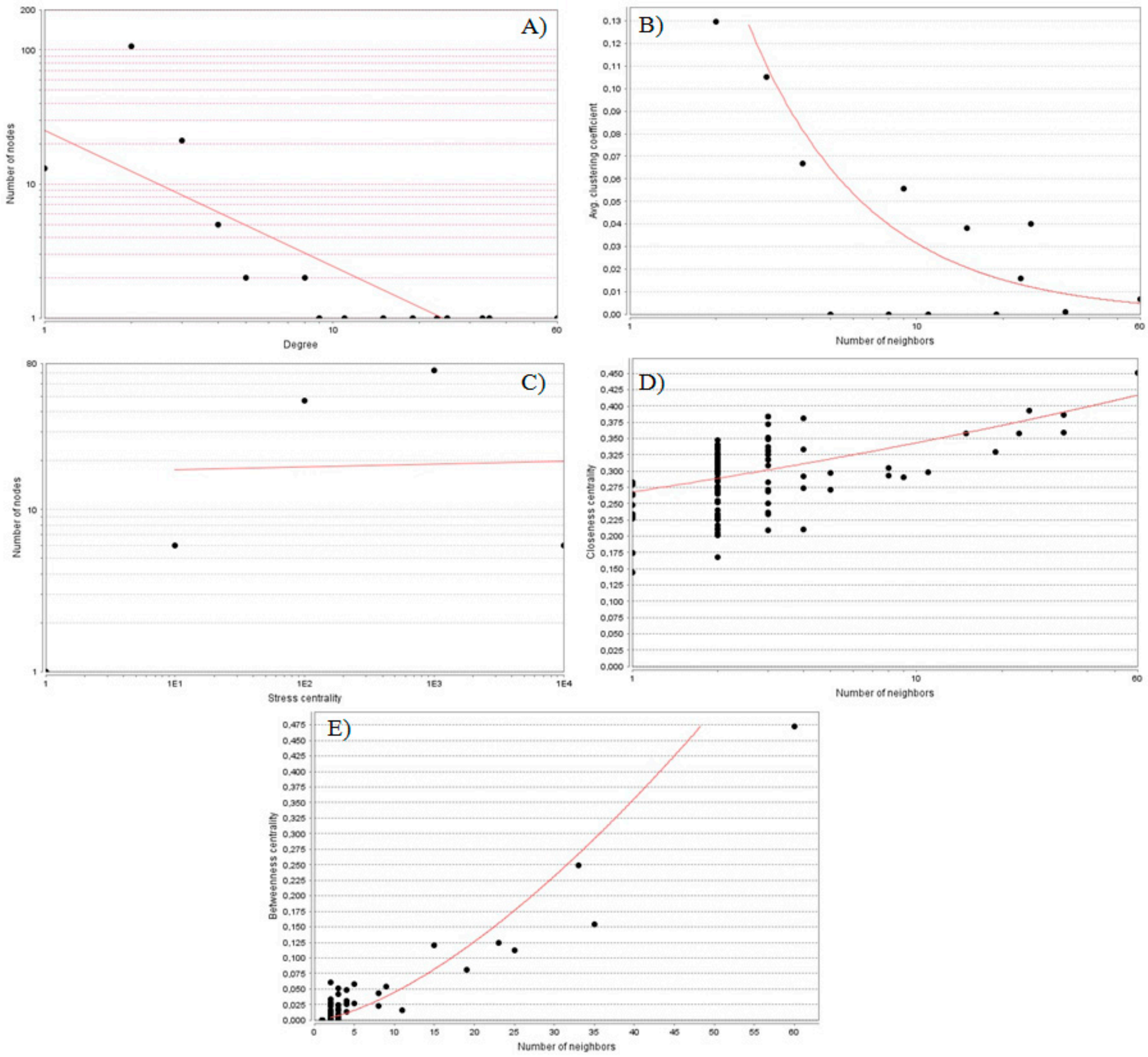

Figure S1. Evaluation of topological properties of network. (A) node degree distribution, (B) average clustering coefficient, (C) stress centrality, (D) closeness cenytrality and (E) betweenness centrality measure.

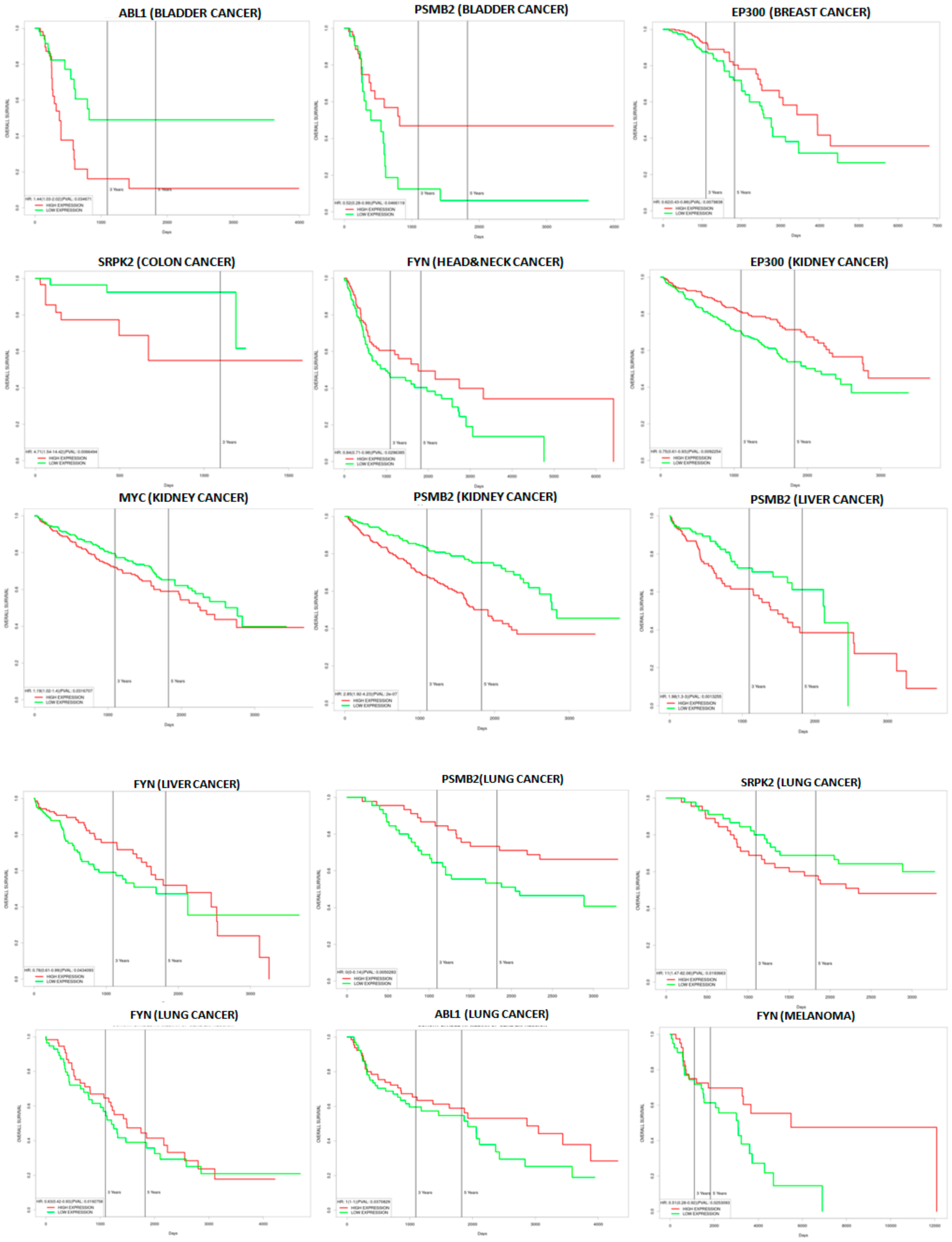
ABL1 (BLADDER CANCER)
PSMB2 (BLADDER CANCER)
EP300 (BREAST CANCER)
SRPK2 (COLON CANCER)
FYN (HEAD&NECK CANCER)
EP300 (KIDNEY CANCER)
MYC (KIDNEY CANCER)
PSMB2 (KIDNEY CANCER)
PSMB2 (LIVER CANCER)
FYN (LIVER CANCER)
PSMB2(LUNG CANCER)
SRPK2 (LUNG CANCER)
FYN (LUNG CANCER)
ABL1 (LUNG CANCER)
FYN (MELANOMA)

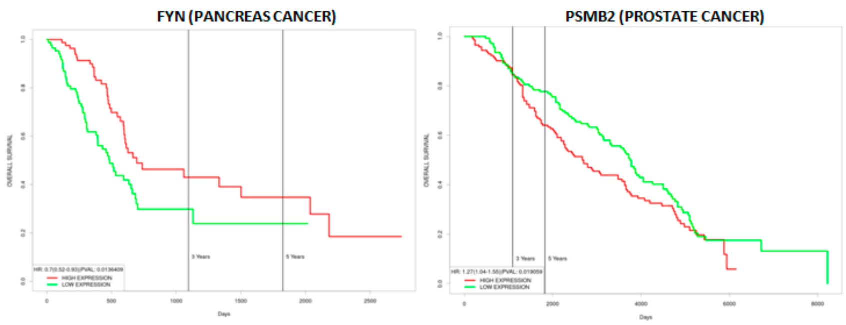

Figure S2. Kaplan-Mayer curves showing the overall survival (expressed in percentage) in solid cancer patients, using PROGgeneV2 online tool, in the case of high and low expression of HUB nodes reported by red and green curves, respectively.

Table S1. List of primer sequences

Gene	Sequence 5'→3'
ABL1	TGGAGATAACACTCTAAGCATAACTAAACCT (31)
	GATGTAGTTGCTTGGGACCCA (21)
DIO1	AGCTTACTCTGGCTTTGCCGA (21)
	TATTACCCGTCTTCTCGCCCA (21)
DIO2	CTTACTCTGGCTTTGCCGAGA (21)
	CAGGATGTTCCGCTTGACTCT (21)
DIO3	GGTAGTTTCCCCCGCTTGTTT (21)
	TTTAGGTGCTGCTTTGAGGCC (21)
EP300	TCCTTTCCATACCGAACC (18)
	GGACAATACGCTCTGATACA (20)
FYN	ATGGAAACACAAAAGTAGCCATAAA (25)
	TCTGTGAGTAAGATTCCAAAAGACC (25)
GPX1	TTATGACCGACCCCAAGCTCA (21)
	ATGTCAATGGTCTGGAAGCGG (21)
GPX2	GGAGAATGAACCCAAGCGAA (20)
	CAGGTTTGTCACAGCCAGTGAT (22)
GPX3	TCTCATCCCATGTCCACCATG (21)
	TGCATCCATTTGTGCCAGG (19)
GPX4	AGAGATCAAAGAGTTCGCCGC (21)
	TCTTCATCCACTTCCACAGCG (21)
GPX6	CAGAAACCCCACCTCACATGA (21)
	TGCCATGACCTGAATGCACT (20)
MSRB1	AGCGGCTGTTGCTCCATAACT (21)
	ATTTCAGCATCACCCACCCTC (21)
MYC	TGAATTAGAATCTCGGGAGTGC (22)
	GAGTGAGACCCCATCTCAGAA (21)
PSMB2	AGAGGGCAGTGGAACTCCTT (20)
	AGGTTGGCAGATTCAGGATG (20)
SELENOF	ATCGGAGGCATGCAGAGAGTT (21)
	TCTGCAATCAGGATCCAGCTG (21)
SELENOH	GGTGGTGGAAGAGTTGAAGAA (21)
	AGGGACACAAAGCTCAGCAT (20)
SELENOI	AAAGGCCAGGTTCCCAGAA (19)
	CAATCCTGCTGCAGTCCAAGT (21)
SELENOK	AATCAATCATCTGCGTGGCC (20)
	TGGTCAGCCTTCCACTTCTTG (21)
SELENOM	TCACGCAGGACATTCCATTCT (21)
	CCTGCACTAGCGCATTGATCT (21)
SELENON	AGGCAGATGCTCATTGTTCCC (21)

	CCCCAAATCCAGATGCAGACT (21)
SELENOO	CGGTTGTGTTGCGTGTAGCTT (21)<br>TGCACTCGAATGTCGTTCCTC (21)
SELENOP	TAGGAGCTGATGCTGCCATTG (21)<br>ATGTTCTCCTCTGCCCGAAGT (21)
SELENOS	CAGCTGCTCGACTGAAAATGC (21)<br>GCATGCTGTCCCACATTTCAA (21)
SELENOT	TCAATCCCACACCATCGATCA (21)<br>ACAACGAGCCTGCCAAGAAAG (21)
SELENOV	GTGGATTCGTCATTTCCCATG (21)<br>TTTGAGTCTGACTGCCATCCC (21)
SELENOW	GTTTATTGTGGCGCTTGAGGC (21)<br>CCATCACTTCAAAGAACCCGG (21)
SEPHS2	CGGCTCGCTTTTGTTCTGAA (20)<br>TCGCGGCTTGTCAATGATC (19)
SRPK2	GCAAAGGACAATGGTGAAGCTGAGG (25)<br>CATCATCATCTTCATCGTCCAGTTGC (26)
TXNRD1	CACAATTGGAATCCACCCTGT (21)<br>GGTTTGCAGTCTTGGCAACA (20)
TXNRD2	AGGACATTTGCTGGTCGAAGC (21)<br>GGAATCCCCTGGAAAAACGTT (21)
TXNRD3	CCTTTCCCAGTTGCTAGTGC (20)<br>GTGCTACACTCTGGGCAACA (20)
VCP	GCCTTGAATGAAGTAGGGTAT(21)<br>GTTGGGTCTGTTGGTTGC (18)
β-actin	TCTGGCACCACACCTTCTACAATG (24)<br>AGCACAGCCTGGATAGCAACG (21)
